# Supplementary figures and images for: An examination of autonomic and facial responses to prototypical facial emotion expressions in psychopathy
Source: PLoS One. 2022 Jul 1;17(7):e0270713. doi: 10.1371/journal.pone.0270713 (PMC9249219; doi:10.1371/journal.pone.0270713)

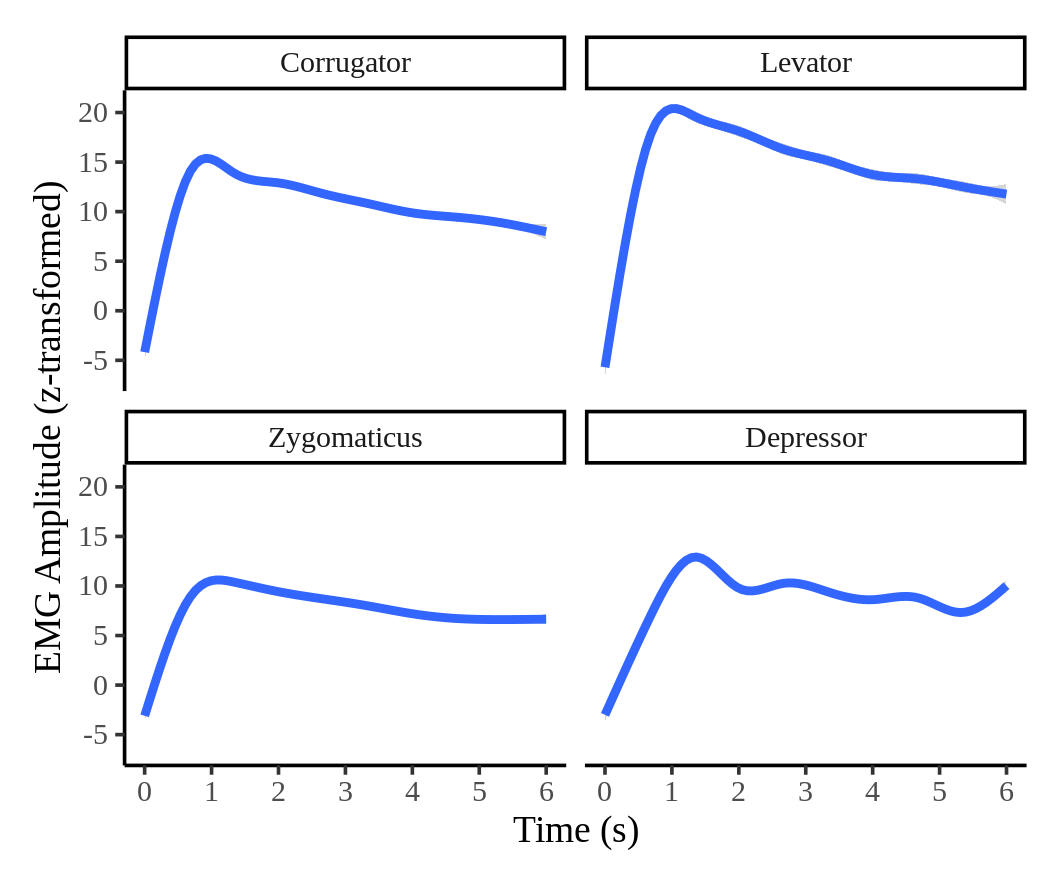

Supplement: S1 Fig — (TIF) [file pone.0270713.s001.tif]
