# Supplementary material for: An examination of autonomic and facial responses to prototypical facial emotion expressions in psychopathy
Source: PLoS One. 2022 Jul 1;17(7):e0270713. doi: 10.1371/journal.pone.0270713 (PMC9249219; doi:10.1371/journal.pone.0270713)
Supplement: S1 Table — (DOCX) [file pone.0270713.s002.docx]

| **S1 Table.** Zero-order correlations among continuous independent variables and dependent variables (*N*=88) | | | | | |  |
| --- | --- | --- | --- | --- | --- | --- |
| **Independent Variables** | | | | | |  |
|  | (1) | (2) | (3) | (4) | (5) | |
| (1) PCL-R Total | - |  |  |  |  | |
| (2) PCL-R Factor 1 | .87*** | - |  |  |  | |
| (3) PCL-R Factor 2 | .93*** | .65*** | - |  |  | |
| (4) Age | -.11 | .02 | -.24* | - |  | |
| (5) Welsh Anxiety | .17 | .10 | .23 | -.01 | - | |
| **Dependent Variables** | | | | | |  |
| Emotion Recognition Accuracy | -.18 | -.18 | -.07 | -.12 | .06 | |
| Valence Rating | .07 | .05 | .05 | -.04 | -.01 | |
| Intensity Rating | -.24* | -.17 | -.20 | .06 | -.06 | |
| *Corrugator* Activity | -.01 | .01 | -.05 | -.15 | .11 | |
| *Levator* Activity | -.11 | -.08 | -.12 | .06 | .21 | |
| *Zygomaticus* Activity | -.05 | -.02 | -.07 | -.07 | .22 | |
| *Depressor* Activity | -.12 | -.06 | -.16 | .00 | .19 | |
| SCR Amplitude | -.24* | -.24* | -.20 | .01 | .08 | |
| Cardiac Deceleration | -.18 | -.13 | -.16 | .47*** | -.06 | |
| *Note: Correlations between each independent variable are provided. Correlations between independent and dependent variables, collapsed across emotion categories except neutral, are provided. Correlations between dependent variables are omitted for brevity.*  *Previous studies have also found a positive relationship between anxiety and corrugator response to emotional images* [1,2]*.*  *SCR = Skin Conductance Response*  ** p < .05, ** p < .01, *** p < .001* | | | | | |  |

**References**

1. Kret ME, Stekelenburg JJ, Roelofs K, de Gelder B. Perception of Face and Body Expressions Using Electromyography, Pupillometry and Gaze Measures. Front Psychol. 2013;4: 1–12. doi:10.3389/fpsyg.2013.00028

2. Smith JC, Bradley MM, Lang PJ. State anxiety and affective physiology: Effects of sustained exposure to affective pictures. Biol Psychol. 2005;69: 247–260. doi:10.1016/j.biopsycho.2004.09.001
