# Supplementary material for: An examination of autonomic and facial responses to prototypical facial emotion expressions in psychopathy
Source: PLoS One. 2022 Jul 1;17(7):e0270713. doi: 10.1371/journal.pone.0270713 (PMC9249219; doi:10.1371/journal.pone.0270713)
Supplement: S1 File — (DOCX) [file pone.0270713.s003.docx]

**Supplemental analyses**

**Eye-tracking**

Psychopathy has been linked to reduced visual fixations on the eye region of facial emotion expressions (e.g., fear expressions) [1]. Participants’ eye gaze during the task was measured with an ASL D6 desk-mounted eye tracker (Applied Science Laboratories, Bedford, MA) at a sampling rate of 60 Hz. Nine-point calibration was performed prior to the experimental task. Fixations, defined as gaze coordinates remaining within 1° visual angle for ≥ 100 ms, were identified offline using automated software. Rectangular areas of interest were drawn around the eye region, mouth region, and the whole face for each video. However, we decided to exclude 17 subjects from eye fixation analyses because of large artifacts in their gaze data. Further, individual trials were excluded if eye tracking failed for > 25% of samples during stimulus presentation. For the remaining 71 participants, this resulted in the exclusion of 12.6% of trials. In all, 32.6% of eye gaze data was excluded from analyses. Calibration issues due to reflection from eyeglasses, participant motion, and issues controlling the lighting in the prison setting likely led to the high exclusion rate. Thus, we do not report results related to eye gaze.

**Deliberate facial muscle movements**

To ensure that each facial muscle’s activity was measured, we instructed participants after the task to deliberately move each muscle in succession. A red square was presented on screen for 6 s, during which time participants were instructed to perform one of four movements: scrunch the eyebrows (*corrugator*), scrunch the nose (*levator*), move the corners of the lips up (*zygomaticus*), or move the corners of the lips down (*depressor*). Each participant completed each muscle movement. EMG data during deliberate muscle movements were processed in the same manner as the EMG data during the facial emotion task. Average, baseline-corrected activity for each participant was calculated by subtracting the average EMG z-score in the 1 s window prior to stimulus onset from the average EMG z-score in the first four seconds of stimulus presentation. Increased muscle activity was observed when participants were instructed to make the appropriate facial movement: *corrugator* (*M* = 11.30, *SD* = 8.22), *levator* (*M* = 15.66, *SD* = 11.23), *zygomaticus* (*M* = 8.28, *SD* = 9.11), and *depressor* (*M* = 8.82, *SD* = 11.22). Average time series for each muscle are presented in S1 Fig.

**Selection of critical muscles for specific emotions**

With EMG measurements of four muscles and six emotion categories, we could have run 24 emotion-specific tests of our facial mimicry hypotheses. We minimized the number of emotion-specific tests by examining only the muscle(s) critical to each prototypical emotion expression. The critical muscles were selected by computing the target person’s facial muscle activity using the Computer Expression Recognition Toolbox (CERT) [2], software that automatically codes 19 action units (AUs) from the Facial Action Coding System [3] for each frame of a video. CERT time series from four AUs, corresponding to the muscles measured via EMG on the participant, were extracted and averaged across 10 stimulus videos for each emotion: AU 4 (brow lowerer), AU 9 (nose wrinkler), AU 12 (lip corner puller), and AU 15 (lip corner depressor). The critical muscles were those whose corresponding AU showed the greatest positive change in CERT activity for each emotion: *corrugator* (AU 4) for anger, *levator* (AU 9) for disgust, *corrugator* (AU 4) and *zygomaticus* (AU 12) for fear, *depressor* (AU 15) for sadness, and *zygomaticus* (AU 12) for joy.

**References**

1. Dargis M, Wolf RC, Koenigs MR. Psychopathic traits are associated with reduced fixations to the eye region of fearful faces. J Abnorm Psychol. 2018;127: 43–50. doi:http://dx.doi.org/10.1037/abn0000322

2. Littlewort G, Whitehill J, Wu T, Fasel I, Frank M, Movellan J, et al. The Computer Expression Recognition Toolbox (CERT). 2011. Available: http://mplab.ucsd.edu/~marni/Projects/CERT.htm

3. Ekman P, Friesen WV, Hager JC. Facial Action Coding System. Ekman P, Friesen WV, Hager JC, editors. Salt Lake City, UT: Research Nexus; 2002.
