# Supplementary material for: An examination of autonomic and facial responses to prototypical facial emotion expressions in psychopathy
Source: PLoS One. 2022 Jul 1;17(7):e0270713. doi: 10.1371/journal.pone.0270713 (PMC9249219; doi:10.1371/journal.pone.0270713)
Supplement: Attachment — Submitted filename: Response to Reviewers - PLOS ONE Second Submission.docx [file pone.0270713.s004.docx]

*Editor/reviewer comments are in italics.*

**Author responses are in bold.**

*Academic Editor*

*I was able to obtain two reviews from colleagues knowledgeable in the areas of psychopathy, emotion, and/or psychophysiology. I reviewed the paper independent of the reviewers. There was broad agreement that the paper had clear themes and focus, and that the experimental methods were fairly rigorous. There was evidence of transparency in the reporting of methods and results, although as noted by Reviewer 1, pre-registration was not included. The reviewers and I noted several limitations regarding the analyses, results, and interpretation of the findings that may be addressed in a greatly revised version of the paper. This is no guarantee of publication though, given the substantial weaknesses noted.*

**We appreciate the opportunity to address the helpful suggestions made by the editor and reviewers. The suggested edits, detailed below, have made the paper clearer and stronger.**

*The physiological data reduction choices need more justification. Different baseline windows were used across EMG and cardiac deceleration, and it is unclear why that is. Reviewer 1 made relevant comments in this regard and provided several other suggestions for you to seriously consider.*

**We have taken Reviewer 1’s suggestion to use the same baseline and response windows for EMG and cardiac deceleration. Based on the reviewer’s suggestion, the baseline window for each measure is 500ms prior to stimulus onset, and the response window is 2-5s after stimulus onset. The baseline window therefore captures facial muscle and cardiac activity in the absence of social stimuli, and the response window captures the period in which the target person (in the stimulus videos) is displaying the prototypical facial emotion expression.**

*I’m having trouble understanding the rationale for not comparing the emotional faces to neutral faces when analyzing valence and intensity rating, as well as physio responses. These comparisons account for between subject variance in general arousal or response tendencies, to get at unique effects of emotional stimuli on response modalities. In some cases in your paper, only descriptive statistics are reported to conclude whether there was sufficient activity or evidence of mimicry. More common analyses involve testing within-subject effects of face category (with specific contrasts of the emotional vs. neutral facets) to examine basic effects, and then including psychopathy scores as between subject factor moderating effects of face category. The absence of these comparisons clouds the potential contribution or meaning of the data.*

**Thank you for this helpful comment. We agree that the suggested analyses offer a clearer picture of participants’ responses to prototypical emotion expressions. We have changed our analyses accordingly. For the dependent variables of valence ratings, intensity ratings, facial mimicry, skin conductance, and cardiac deceleration, we report within-subject comparisons of emotion vs. neutral trials. These analyses establish whether prototypical emotion expressions, rather than faces per se, elicit arousal and facial muscle activity. We then report mixed effects models, which include psychopathy scores as a between-subjects factor moderating the within-subjects effect of emotion vs. neutral trials.**

**We note that we did not compare emotion vs. neutral trials for the dependent variable of emotion categorization accuracy. The editor omitted this dependent variable when suggesting comparisons of emotion vs. neutral trials, and we agree that such comparisons are not warranted. Prior research supports our current analyses, in which we test accuracy across as well as within emotion categories (see Dawel et al., 2012). However, we have added independent-sample t-tests to demonstrate that participants performed the emotion categorization task better than chance, according to the editor’s suggestions.**

*Reviewer 2 makes important points about the reporting of results and ambiguousness in interpretation. I agree that in general, if you are using NHST, you should stick to the p values you determine. The interpretation of trend level p values is problematic, and when you report results both with and without outliers, it is unclear which results we should rely on and what is the rationale for the choice. What were the planned analyses in terms of outlier and covariate inclusion criteria? This can help determine which results are primary in your interpretation. Please also include effect size estimates to understand the impact and potential meaningfulness of the results. If effects are small, indicate whether this size of effects can still provide important information and how.*

**We no longer describe results as “trend-level” and we have removed interpretations of such results from the manuscript. Additionally, we now report only analyses with covariates and report regression coefficients as effect size estimates for each model.**

**We performed outlier analyses for each of the models. However, removing outliers from the models no longer affects the significance of our results. In other words, our results are significant with or without outliers in the model. We have therefore omitted outlier analyses from the current submission.**

*Neither reviewer (and I agree) found the exploratory analyses particularly compelling or meaningful, especially due to the inconsistent manner of the groupings and small sample sizes. Instead, it would have been more straightforward and consistent with theory to examine factor or facet level relationships with mimicry and autonomic arousal to emotion faces. The primary focus of the paper on total psychopathy, despite persistent evidence of differential relationships between specific psychopathic traits and emotional functioning is confusing. As noted by Reviewer 2, there are choices in hypotheses and analyses that are not consistent with prior work or theory. This should be noted explicitly.*

**Per the editor’s suggestion, we have moved the analyses of PCL-R Factor 1 and Factor 2 to the main text. We hypothesized relationships between behavioral and physiological responses and Factor 1, but not Factor 2.**

**We appreciate the editor’s and reviewers’ concerns about the exploratory subtype analyses. These concerns questioned the theoretical rationale, the methods used to derive the subtype groups, and the power of the analyses. To address these concerns, we have removed these exploratory analyses from the current submission. Since the hypothesis behind the exploratory analyses is worthwhile, we have added a suggestion for future studies (with sufficient power) to perform these analyses (p. 26):**

**“Alternatively, heterogeneity among high psychopathy individuals might help to explain the present null findings regarding sympathetic arousal. Future studies might examine whether two subtypes of psychopathy, distinguished by low versus high levels of trait negative affect and anxiety (78–82), display different patterns of sympathetic arousal to socioemotional stimuli such as faces. Though the current sample of high psychopathy individuals (PCL-R ≥ 30) was too small to test the hypothesis (n = 30), prior work has reported reduced sympathetic arousal to socioemotional stimuli specifically among high psychopathy individuals with low levels of negative affect and anxiety (23,24).”**

*When submitting your revision, we need you to address these additional requirements.*

*1. Please ensure that your manuscript meets PLOS ONE's style requirements, including those for file naming. The PLOS ONE style templates can be found at*

*https://journals.plos.org/plosone/s/file?id=wjVg/PLOSOne_formatting_sample_main_body.pdf and*

*https://journals.plos.org/plosone/s/file?id=ba62/PLOSOne_formatting_sample_title_authors_affiliations.pdf*

**We adjusted the manuscript style and file names to be in line with PLOS ONE’s style requirements.**

*2. Please provide additional information regarding the considerations made for the prisoners included in this study. For instance, please discuss whether participants were able to opt out of the study and whether individuals who did not participate receive the same treatment offered to participants.*

**We have added this information to the Participants section (p. 6): “Participants were informed that their participation was voluntary and would not affect their institutional status.”**

*3. We note that you have stated that you will provide repository information for your data at acceptance. Should your manuscript be accepted for publication, we will hold it until you provide the relevant accession numbers or DOIs necessary to access your data. If you wish to make changes to your Data Availability statement, please describe these changes in your cover letter and we will update your Data Availability statement to reflect the information you provide*

**We understand and appreciate this policy. We plan to provide the relevant accession numbers or DOIs at acceptance.**

*4. We note that your paper includes detailed descriptions of individual patients/participants. As per the PLOS ONE policy (http://journals.plos.org/plosone/s/submission-guidelines#loc-human-subjects-research) on papers that include identifying, or potentially identifying, information, the individual(s) or parent(s)/guardian(s) must be informed of the terms of the PLOS open-access (CC-BY) license and provide specific permission for publication of these details under the terms of this license. Please download the Consent Form for Publication in a PLOS Journal (http://journals.plos.org/plosone/s/file?id=8ce6/plos-consent-form-english.pdf). The signed consent form should not be submitted with the manuscript, but should be securely filed in the individual's case notes. Please amend the methods section and ethics statement of the manuscript to explicitly state that the patient/participant has provided consent for publication: “The individual in this manuscript has given written informed consent (as outlined in PLOS consent form) to publish these case details.”*

**In an email exchange with an editorial assistant, Olympia Duman, the first author ascertained that this note was referring to images of faces presented in Figure 1. We note that the images in Figure 1 were taken from the stimuli set, the Amsterdam Dynamic Facial Expression Set. The images do not portray individual participants. Nor do we present detailed descriptions of individual participants elsewhere in the manuscript. We amended the Figure 1 legend to clarify that the images portray videos from the stimuli set.**

*Reviewer 1*

*This intriguing study provides a multimodal test of the impact of facial expressions on those with psychopathic traits, going beyond mere facial mimicry to examine autonomic functioning in addition. However, the critical subgrouping did not proceed in the way the authors analyzed their data, making it challenging to understand the contribution of this work to the psychopathy literature. Further aspects of the data reduction bear scrutiny to understand the panoply of surprising null results.*

**We thank the reviewer for the thoughtful suggestions below, which have made the paper’s contribution to the psychopathy literature clearer.**

*INTRODUCTION*

*The introduction does a nice job of setting up the rationale for the study. However, I couldn’t find the link to the registration that would allow me to verify which analyses were confirmatory and which were exploratory. What is the link to that registration?*

**The study was not preregistered.**

*Also, to what extent are PCL-R factors differentially associated with facial recognition deficits?*

**We have added a citation to the introduction to address this question (p. 3):**

**“In particular, a diminished capacity to categorize (or “recognize”) prototypical facial emotion expressions is thought to be associated with the deceitful interpersonal and callous affective features of psychopathy (2).”**

*METHOD*

*Why were seven participants missing Welsh anxiety data? Were the profiles uninterpretable for some reason? If so, the specific validity scales for those exclusions should be given. Or were the data just missing? In either case, if the MPQ data were valid for those participants, some kind of imputation procedure should be used to impute these scores. Otherwise, the participants analyzed in the covariate analyses are not the same as those in the whole sample (eliminating almost 10% of the whole-sample participants), which distorts the meaning of the covariate analyses relative to those in the whole sample.*

**Seven participants simply failed to complete and return the Welsh Anxiety Inventory. We have taken the reviewer’s helpful suggestion to multiply impute these missing values, along with several other missing data points. The details of our multiple imputations procedure can be found on p. 11:**

**“In total, 33 out of 88 participants (37.5%) had incomplete data. We therefore imputed missing data to avoid potential bias in the analyses. Ten imputed data sets were derived from a bootstrapped expectation maximization algorithm (the 'amelia' package in R; 50). All independent and dependent variables were included in the multiple imputation algorithm. Statistical inference was made based on analyses pooled across the ten imputed data sets (using the ‘mitml’ package in R). The imputed data allowed for the inclusion of participants who were missing data for the following reasons: participant failed to complete the WAI (n = 7), omitted items prevented calculation of PCL-R Factor 2 score (n = 7), facial hair prevented EMG measurement of depressor activity (n = 22), artifactual noise prevented observation of SCRs (n = 1) and heartbeats (n = 1), and irregular heartbeat prevented observation of reliable stimulus-evoked cardiac deceleration (n = 1).**

*What were the PEM, NEM, and CON scores on the MPQ to be placed in Table 1? T scores would be preferable to report for such analyses to make the interpretation easier, as in the IQ measure.*

**The reviewer’s question was in regards to the exploratory analyses in the previous submission, which used cluster analyses of the MPQ subscales (PEM, NEM, CON) to derive subtypes of high psychopathy individuals. We have removed these exploratory analyses from the manuscript in response to reviewers’ concerns about the rationale and power of the analyses. In the current submission, we report sample characteristics for all relevant assessments. This does not include MPQ scores, since these scores are not relevant to the analyses in the manuscript.**

*The term “Caucasian” should be deprecated in referring to White people, as that particular term has a long (and long-criticized) history implying racial superiority of individuals so labeled: https://www.ncbi.nlm.nih.gov/pmc/articles/PMC1444385/pdf/bmjcred00490-0048.pdf*

**Thank you for this recommendation. We have changed the instance of “Caucasian” to “white.”**

*A 25 kΩ impedance is relatively high compared to published guidelines; why was such a high level of impedance considered acceptable in this study?*

**Our EMG impedance threshold was higher than some published guidelines. For example, Tassinary et al. (2007) recommend an impedance threshold of 10 kΩ. We have added this as a methodological limitation (p. 26):**

**“The EMG impedance threshold, which was higher than some published guidelines (86), is another methodological limitation of the study.”**

*Why was a 60 Hz notch filter used in this dataset? Was there evidence of line noise? Using a 60 Hz filter without line noise can introduce noise and ripples into the data that would not otherwise exist.*

**We observed evidence of 60 Hz line noise. This noise was present because we were unable to prevent electrical noise in the testing room at the prison.**

*If the facial stimulus started changing 1 s after the face’s onset and reached peak expression at 3 s – 4 s, why was not the EMG scoring window from 2 s – 5 s to account for the cited 1 s delay in EMG onset once an expression is evident and the stimulus parameters described above? Or was this procedure meant just to examine psychopathy’s relationship to any facial stimulus (given the ramp-up in the supplementary figure) rather than mimicry specifically?*

**We have changed our analyses based on this helpful suggestion. Originally, we modeled the EMG response 1-3s after stimulus onset based on prior work showing that facial mimicry (measured by EMG) can be observed within 1s after presentation of a prototypical facial emotion expression (Dimberg & Thunberg, 1998). However, we agree that this response window omitted the peak of the target person’s facial muscle activity (3-4s after stimulus onset). Therefore, we recalculated EMG responses using the suggested window 2-5s after stimulus onset.**

*The SCR data analysis was clever. I assume the canonical function was estimated for the entire 6 s of stimulus presentation? How long was the ratings presentation?*

**Yes, the canonical function was estimated for the entire 6s of stimulus presentation. The ratings screens were self-timed and varied by participant. The ratings screens were presented for at least 3.6s, as can be seen from the range of presentation times in Figure 1.**

*For the cardiac deceleration data, would it not make sense to have a similar analytical window as the EMG data proposed above? That way, decelerations not due to a particular facial expression would be excluded. Or were the researchers interested only in the deceleration to human faces, irrespective of their portrayed emotional expression?*

**We have taken the reviewer’s suggestion to change the response window for the cardiac deceleration analyses. The response window is now the same as that for the EMG analyses (2-5s after stimulus onset), thus capturing the onset and peak of the prototypical facial emotion expression.**

*I liked the authors’ justification for choosing only target muscles for investigating particular target emotions, as those analytical decisions follow well from the theoretical literature. In-text citations would help defend those even more strongly outside of the supplementary materials.*

**We have added Simon et al. (2008) as an in-text citation to support our selection of target muscles critical for each prototypical emotion expression (p. 12):**

**“The number of emotion category-specific tests for facial mimicry was minimized by testing only the muscle(s) critical to each prototypical emotion expression (51): corrugator for anger, levator for disgust, corrugator and zygomaticus for fear, depressor for sadness, and zygomaticus for joy.”**

*Were covariates centered in the analyses involving them? Not doing so can eliminate main effects of interest.*

**In our previous submission, covariates of age and anxiety were not mean-centered. In the current manuscript, covariates were mean-centered according to the reviewers’ guidance. We note that, because we did not model interactions between our primary independent variable (PCL-R Total) and the covariates, changing the mean of the covariates did not change the main effect of interest.**

*From the authors’ exploratory analyses described in the introduction, it seems that the Welsh anxiety x PCL-R total score interaction should have been entered – or was it, and I just misunderstood the terms in the statistical model?*

**We have removed the exploratory analyses of psychopathy subtypes from the current submission in response to reviewers’ concerns about the rationale and power of the analyses. The reviewer is right to note that prior studies have analyzed subtypes of psychopathy with an interaction of anxiety and psychopathy scores. However, our lab uses cluster analyses to derive subtypes of high psychopathy individuals (Hicks et al., 2004; Dargis & Koenigs, 2018).**

*The clustering approach described in the supplementary analyses does not identify specific subtypes of individuals with psychopathy. Instead, it will divide the overall sample into two groups across all levels of the PCL-R.. Also, it appears that two participants were excluded from the high psychopathy group (n = 30) when the subtype analyses were conducted (n = 28); why were they missing? I assume these were the two participants for whom MPQ data were not available.*

**We appreciate the opportunity to clarify the methods for our exploratory analyses, which have been removed from the current submission. We derived low-NA and high-NA groups from a cluster analysis of a larger sample of high psychopathy individuals (PCL-R ≥ 30), which has been described by Weaver et al. (2021). The *n*=30 high psychopathy participants in the present sample were a subset of this larger set of high psychopathy individuals. The subtypes were created in a larger sample of high psychopathy individuals, not across all individuals in the present study’s sample (*N*=88).**

**The reviewer is correct – the two participants missing from our exploratory analyses never completed or returned their MPQ forms to the research team.**

*Finally, k means cluster analyses do not have strong stopping rules; model-based cluster analyses would be preferable to examine whether two groups or more are optimal in these data with the ability to quantify the probability of each participant’s membership in each cluster.*

**The reviewer makes a good point about the stopping rules of k-means cluster analysis. However, we have removed the exploratory analyses of psychopathy subtypes (which used k-means clustering) from the current submission.**

*It may be preferable to describe the subtype variables as reference-coded coded rather than dummy variables to prevent potentially ableist interpretations of the language. The notion of “reference coding” also makes it clearer that a specific group is the reference to which all others are being compared.*

**We appreciate the reviewer’s suggestion. We have changed instances of “dummy” to “reference-coded” in the relevant section, which now appears in the supplemental materials.**

*RESULTS*

*In general, p values should be reported to at least 3 decimal places.*

**We now report p values with 3 decimal places.**

*What was the critical value of Cook’s distance that was used to identify outliers?*

**Because we changed our analytic strategy based on reviewer feedback, removing outliers from the models no longer affects the significance of our results. In other words, our results are significant with or without outliers in the model. We have therefore omitted outlier analyses from the current submission.**

**To answer the reviewer’s question directly, we used the following critical value of Cook’s distance: 4/(N-P) = 4/(88-4) = 0.0476.**

*What were the effect sizes for the various F statistics that were computed?*

**We now report regression coefficients as effect size estimates for each of the analyses.**

*The lack of significant results in the EMG analyses may result from the covariates being uncentered and the suboptimal windows used to score data.*

**Thank you for raising this possibility. Changes to our analytic strategy for the EMG data – centering covariates, optimizing the response windows, and contrasting emotion vs. neutral trials – resulted in null results that paralleled our original analyses.**

*The subtype analyses appear inappropriate given that the subtypes were created across all participants in the dataset, yet only those in the high psychopathy were divided this way. It would be more appropriate to maintain the splits across intermediate and low groups as well.*

**We have responded to this concern in more detail above. Although the exploratory analyses have been removed from the current submission, we can clarify that the subtypes were created in a larger sample of high psychopathy individuals (described by Weaver et al., 2021), not across all individuals in the present study’s sample (*N*=88).**

*DISCUSSION*

*Could the lack of surprise faces have been one reason for the lack of psychopathy-related results compared to previous meta-analyses?*

**This is an interesting suggestion. The Dawel et al. (2012) meta-analysis found that psychopathy was related to reduced categorization (or “recognition”) accuracy both across emotional expressions, and specifically for prototypical expressions of the categories of fear, happiness, sadness, and surprise (but not anger or disgust). However, our analyses showed no effect of psychopathy across emotional expressions or for specific emotion categories. The lack of inclusion of prototypical surprise faces likely did not drive the null results.**

**We have expanded our discussion of the possible explanations for these null findings (p. 23):**

**“We have considered the following explanations for the present null findings. First, a ceiling effect in the current study (90% categorization accuracy for all emotions across participants) may possibly have prevented the observation of a deficit in relation to psychopathy. Second, omitting prototypical surprise expressions from the present study’s stimuli might have reduced the observed relationship between psychopathy and emotion categorization accuracy. However, this explanation seems unlikely, given that meta-analyses found categorization deficits for emotion categories that were included in the present study’s stimuli, including fear, sadness, and happiness (2,3).”**

*I was surprised that no mention of the characteristics of the two groups of participants were discussed anywhere in the text. Was it only NEM on which they differed, or did they differ on other MPQ variables? And were the domains of the MPQ used or the primary trait scales?*

**We understand the reviewer’s concern. We have not added the requested group characteristics because we have removed the exploratory subtype analyses from the current submission. We report sample characteristics for all assessments that were relevant to our analyses.**

*Reviewer 2*

*This manuscript reports results from a study examining the relationship between psychopathic traits (as measured by PCL-R) and autonomic and facial mimicry responses to dynamic facial emotion expressions, in addition to emotion categorization accuracy and valance and intensity judgments. The idea for the study and the methodological design was impressive and would constitute an addition to the scant and inconsistent literature in this area. It appears that the authors largely found null results, at least for the analyses described in the manuscript, with the exception of significant results for the exploratory analyses where participants with high scores on the PCL-R (greater than or equal to 30) were divided into “high-NA” and “low-NA” subgroups through cluster analysis of the MPQ-BF scales. The methodology and study design are a strength of the manuscript, and the null results are interesting and worthwhile of discussion on their own. I commend the authors for clearly delineating between the a priori and post-hoc/exploratory analyses that they conducted. Unfortunately, the authors’ discussion of their results, particularly the questionable conclusions drawn from the data and insufficient justification provided for the chosen exploratory follow-up analyses, was a significant weakness of the manuscript. Based on the way that the statistical results are interpreted and described in the manuscript, it is not suitable for publication in its present form, as it does not meet criteria for publication (particularly, Criterion 4: “Conclusions are presented in an appropriate fashion and are supported by the data”). The merit of the study methodology is important, and should the authors revise the document to reflect the feedback below, the manuscript might be suitable for publication and constitute a valuable addition to the published literature in this area.*

**We are grateful for the reviewer’s helpful suggestions. We have revised the manuscript to address the reviewer’s concerns, particularly the concern that the data support the conclusions.**

*Major Concerns*

*(1) Questionable interpretation of statistical results.*

*a. In the abstract, discussion, and conclusion, the statistical results appear to be inappropriately described/interpreted.*

*b. For example, in the abstract, references are made to “trend level” results and a conclusion is stated that: “These findings provide marginal evidence for reduced intensity ratings of and sympathetic arousal to prototypical facial emotion expressions in high psychopathy individuals.” This is a generous interpretation of the findings at best, and verges on questionable practices at worst.*

**Thank you for the opportunity to address this concern. We no longer describe results as “trend-level” or “marginal evidence,” and we have removed interpretations of such results from the manuscript.**

*1. The findings for reduced intensity ratings did not survive removal of outliers. Per Figure 2, it is notable that one outlier on the very low end of psychopathy scores had an averaged intensity rating of 6 out of 6 across emotions. The results of this analysis with outliers included are reported as significant, and then reported as “trend level” once the outliers were removed and the p-value became greater than 0.05. If it is meaningful to include the results with outliers not removed, then the authors will need to explain the potential impact of the extreme outliers (particularly the one described on the low end of psychopathy scores) on the results.*

**We understand the reviewer’s concern about the meaning of results that change depending on the inclusion of outliers. Because we changed our analytic strategy based on reviewer feedback for the current submission, removing outliers from the models no longer affects the significance of our results. In other words, our results are significant with or without outliers in the model. We have therefore omitted outlier analyses from the current submission.**

*2. The findings for reduced SCR were not significant when covariates were included. In the Data Analysis section (lines 189-195), it specifically states that the covariates of age and anxiety have been found to impact SCR, hence the inclusion of those variables as covariates. If the results of the analysis without covariates is meaningful, the authors need to provide an explanation of the impact that including vs. excluding those covariates may have had on the results.*

**We agree that the SCR results from the previous submission required further clarification, given that results differed when covariates were excluded from the model. We now only report models with covariates included. We also changed our analyses based on suggestions made by the academic editor. The SCR analyses now include a contrast between emotion vs. neutral trials, in order to control for responses to faces in general. Thus, the SCR results in the current manuscript are uniformly null.**

*2) Insufficient justification for follow-up exploratory analyses.*

*a. First, justification will need to be provided for the choice to conduct exploratory follow-up analyses on the “trend level” results in the first place. Why not just describe the null results as null results and leave it there? Null results are still meaningful and important, particularly when the study methods appear rigorous.*

**Following the reviewer’s suggestion, we no longer describe results as “trend-level.” We describe null results as null and discuss potential explanations for the null findings. We agree that null results are meaningful and important.**

*b. Second, justification will need to be provided for the choice of exploratory follow-up analyses. Specifically, why look at low and high NA? Beyond the fact that such subtyping has been completed in prior studies, why is this particular break-down supported by theory and expected to be related to SCR and emotion intensity ratings?*

**We have removed the exploratory analyses of psychopathy subtypes from the current submission in response to reviewers’ concerns about the rationale and power of the analyses. We note that Karpman (1941) and others hypothesized two subtypes of psychopathy distinguished by levels anxiety and negative affect. Historically, a variety of methods have been used to identify the subtypes of high psychopathy individuals, including a median split of anxiety scores (Newman et al., 2005), the Antisocial Personality Questionnaire (Blackburn et al., 2008), and cluster analyses of the Multidimensional Personality Questionnaire (Hicks et al., 2004; Dargis & Koenigs, 2018) and anxiety scores (Kimonis et al., 2011). These subtyping methods share the theoretical underpinning that two subtypes of psychopathy differ in affective processing. Thus, our exploratory analyses examined differences between the subtypes in SCR and intensity ratings.**

*1. Table S2 in the Supplemental Materials shows that SCR amplitude was negatively correlated (r = -.024, p < .05) with both PCL-R Total score and PCL-R Factor 1 (which is interesting, considering the interpersonal/affective factor would be expected to be related to arousal to affective stimuli). Table S7 shows that Factor 1 did not show significant relationships with SCR amplitude, especially when corrected for multiple testing, but what about looking at the facet level (i.e., interpersonal vs. affective vs. impulsive vs. antisocial facets)? The authors need to explain why/how they decided on the high vs. low-NA psychopathy subtyping rather than other potential post-hoc analyses that could have been completed and may have theoretical backing.*

**We have removed the exploratory analyses of psychopathy subtypes from the current submission. To limit the number of tests reported in the study, we report analyses of the two PCL-R factors (interpersonal/affective, lifestyle/antisocial) but not the four PCL-R facets (interpersonal, affective, lifestyle, antisocial).**

*2. The authors will also need to explain why they looked at high and low-NA only in the “high psychopathy” group rather than looking at high and low-NA at all levels of psychopathic traits and must discuss the impact that the choice may have had on the results. For example, how can the reader tell whether the findings of reduced SCR in the low-NA high psychopathy group are not related to low-NA more broadly?*

**This is a great question about our exploratory analyses, which have been removed from the current submission. Had the exploratory analyses been sufficiently powered, we could have shared results that addressed this question. Specifically, we clustered the low psychopathy group into high-NA and low-NA groups. SCR amplitude did not differ between these two groups, suggesting it was not an effect of low-NA more broadly.**

*i. In drawing conclusions from these findings, it would be helpful for the authors to explain to the reader what the low and high-NA clusters are (as constructs) when anxiety is controlled for as a covariate, particularly when interpreting the results shown in in Table 3 (p. 15).*

**The reviewer makes a good point that our exploratory analyses, which have been removed from the current submission, needed further clarification, given that the covariate of anxiety likely removed some variance accounted for by the subtype groups. Had the exploratory analyses been sufficiently powered, we could have addressed this comment by reporting significant group effects with and without covariates.**

*Minor Concerns*

*(1) Psychopathy appears to be largely presented as a unitary construct through the reliance on Total PCL-R score and limited discussions of the factor and facet structure. Adding some more discussion about factor/facet-level results (and maybe the relationship between the factors/facets and the high-NA and low-NA subtypes) would improve the paper. From the references, it is clear that the low- and high-NA subtypes have shown associations with aspects of “primary” and “secondary” psychopathy. Adding information like that to the text would help contextualize these subtypes to the reader and would tie it in with the broader literature on psychopathy.*

**Per the reviewer’s suggestion, we have moved the analyses of PCL-R Factor 1 and Factor 2 to the main text. We hypothesized relationships between behavioral and physiological responses and Factor 1, but not Factor 2. However, we removed the exploratory subtype analyses from the current submission.**

*(2) The write-up is light on discussion of theory and appears to rely on “novelty” to support the publication of the study and its findings, which is not a sufficient reason to publish anything. Just because something is new does not necessarily mean that it is worthwhile. On the other hand, the theoretical basis, and the role of this study as a “theory test” to move the field forward would be far more compelling.*

**We agree that there is a need to derive hypotheses from existing theory. The relevant existing theory is Blair’s Integrated Emotion Systems Theory, which would predict reduced categorization accuracy for prototypical expressions of fear, sadness, and joy (Blair, 2005). However, meta-analyses have found reduced categorization accuracy for a broader range of emotion categories (Dawel et al., 2012). Therefore, we based our primary hypotheses on the meta-analytic findings rather than prior theory. We have introduced other relevant theoretical perspectives, such as embodied simulation accounts and the many-to-many mapping of facial expressions to emotion categories, in order to justify our analyses.**

*a. Regarding theory-based hypotheses, the hypothesis about valence ratings (based on one study done in an all-female sample) is a bit weak, particularly since you have an all-male sample. Obviously don’t change up your hypotheses post-hoc, but it is a point of illustration. You could have made that hypothesis based on theory, and it would have been a stronger one.*

**The reviewer has brought up a useful example. To clarify our rationale for examining valence ratings in the first place, we have added a paragraph discussing the shifting science of facial expressions in neurotypical people (p. 5):**

**“Importantly, there is reason to scrutinize the assumption that any person, irrespective of psychopathy, can accurately read emotions from faces. This assumption stemmed from cross-cultural research that claimed to identify universal “basic emotions,” which we call “emotion categories,” that correspond to unique facial configurations, which we refer to as “prototypical emotion expressions” (32). Accumulating evidence suggests that people do not reliably smile when happy, scowl when angry, or frown when sad (33,34). There is not a one-to-one mapping between a person’s emotional state and facial configuration. Moreover, facial expressions also appear to convey social information (35) and information about two dimensions of a person’s affect, namely valence and arousal (33,36). To characterize the callousness of people with psychopathy, a large body of research has examined how they perceive emotion categories from faces (2–4,37,38), but only one study has examined how they perceive affect from faces (39). Women high in psychopathy perceived another person’s prototypical joy expressions as less positive, compared to women low in psychopathy. In the current study, we attempted to replicate and extend this finding. The shifting science of how neurotypical people use facial configurations to express and perceive affect and emotion could lead to new insights into high psychopathy people’s callous lack of empathy.”**

*(3) Group Ns should be included in all tables/figures consistently.*

**We now report relevant *N*s in each table and figure legend.**

*(4) Where is the power analysis? The group sizes are small, especially for the subgroup analyses with low- and high-NA. The small group size is briefly mentioned as a limitation (p. 21) but mention of statistical power should be presented along with the results (although, see my concern above that the choice to do the exploratory analyses on “trends” in data).*

**We understand the reviewer’s concerns about the power of the exploratory analyses. We have removed the exploratory subtype analyses from the current submission.**

**In addition, we have added a statement regarding power analyses (p. 12):**

**“According to power analyses, the above tests had 80% power to detect a small effect size (f^2^ ≥ 0.02 and < 0.15; 59).”**

*(5) Further, where are the effect sizes? It is laudable that the authors plan to make the data available upon publication, but the reader should not have to run the analyses themselves in order to determine the effect sizes for these “trends.”*

**We now report regression coefficients as effect size estimates for each of the analyses.**
