# Supplementary material for: An examination of autonomic and facial responses to prototypical facial emotion expressions in psychopathy
Source: PLoS One. 2022 Jul 1;17(7):e0270713. doi: 10.1371/journal.pone.0270713 (PMC9249219; doi:10.1371/journal.pone.0270713)
Supplement: Attachment — Submitted filename: Response to Reviewers - PLOS ONE Third Submission.docx [file pone.0270713.s005.docx]

*Reviewer comments are in italics.*

**Author responses are in bold.**

*Reviewer #1*

*In reading over this revision, I am impressed with the authors’ attention to the details of the reviewers’ comments. In all, this work provides an important multimodal assessment of reactivity to facial emotional displays in a sample of incarcerated men using multiple methods of displaying facial expressions and various methods of measuring reactivity. It also then shows how psychopathy is not associated with these forms of reactivity. I have a few remaining clarifying questions for this submission.*

**We appreciate the positive comments in addition to the reviewer’s helpful suggestions on this and prior drafts.**

*Within the introduction, the literature summary on page 4, paragraph 2 made me wonder the degree to which reactivity to facial expressions differs in incarcerated samples relative to non-incarcerated samples broadly speaking. However, this is more a point of curiosity that arises based on the overall null results that might contrast against those obtained in non-incarcerated samples. Wilson et al. (2011) suggests there are no meaningful differences between non-incarcerated and incarcerated samples, whereas Dawes et al. (2012) included only two non-incarcerated samples, making that kind of moderator analysis impossible to perform.*

**We agree that this is an interesting empirical question. We have added this point to the Discussion (p. 23-24):**

**“Third, the origin of the current sample (a forensic population) may have influenced the null results. Once again, this explanation seems unlikely, given that the origin of the sample (forensic vs. community) does not appear to influence the relationship between psychopathy and emotion categorization accuracy (3).”**

**Reviewer 2 asked a similar question about the role that the forensic sample played in the null facial mimicry results. We added the following point to the Discussion (p. 26-27), which is also relevant to Reviewer 1’s question:**

**“It is possible that facial mimicry is less pronounced among incarcerated samples than community samples. In general, incarcerated samples differ from community samples along dimensions that may affect mimicry in response to white faces, including having higher prevalence of mental illness (83) and traumatic brain injury (84), greater proportion of racial and ethnic minority groups (85), and lower socioeconomic status (86). However, Künecke and colleagues found no facial mimicry differences between incarcerated and non-incarcerated individuals (13).”**

*In the method, it would be helpful to add a sentence describing precisely the evidence of 60 Hz line noise that required notch filtering. For example, a diagram of a prototypical trial with visible 60 Hz noise or the output of a fast Fourier transform showing a peak at 60 Hz could be added to the supplemental materials, or a verbal description of either of these results in the method could do the same.*

**We have added a sentence describing the 60 Hz line noise that required notch filtering (p. 9):**

**“A 60 Hz notch filter was applied because a fast Fourier transform showed a peak at 60 Hz due to electrical noise.”**

*When describing in the discussion that a relatively high impedance is a limitation, it would be important to do more than just state it as such. There should be a sentence or two describing why it is a limitation to the study (e.g., impedances that high may prevent reading the underlying signal; they may also inject noise into the data).*

**We have added a sentence describing why high impedance is a limitation of the study (p. 27):**

**“Although we were able to detect deliberate facial muscle movements (see S1 File), high EMG impedance could have prevented detection of more subtle muscle movements.”**

*Reviewer #2*

*This manuscript reports results from a study examining the relationship between psychopathic traits (as measured by PCL-R) and autonomic and facial mimicry responses to dynamic facial emotion expressions, in addition to emotion categorization accuracy and valance and intensity judgments. Despite the null findings for most analyses described in the manuscript, the idea for the study and the methodological design is impressive and will constitute an addition to the scant and inconsistent literature in this area. The methodology and study design are a strength of the manuscript, and the null results are interesting and worthwhile of discussion. The changes made to the manuscript in response to editor and reviewer comments on the initial submission have brought the document into alignment with the criteria for publication. I have provided a few minor revision suggestions for the authors, with the first point (transparency about a priori and post-hoc data analysis decisions) being the most essential to address prior to publication.*

**We appreciate the author’s helpful suggestions on this and prior drafts.**

*1) Since the analyses were changed in response to reviewer/editor feedback, transparency is essential. It will be important to include some mention that the analyses (and thus, associated hypotheses) are post-hoc.*

**We thank the reviewer for the suggestion to make our report more transparent. We have added parenthetical notes to the Methods section (p. 9, 11) about which analytic choices**

**were recommended by reviewers.**

**However, we would disagree that our hypotheses were post-hoc. Our hypotheses – reduced emotion categorization accuracy, more neutral valence ratings of joy, lower intensity ratings for all emotions, reduced facial mimicry, and reduced autonomic arousal – were the same in our original submission to PLOS ONE and in our current submission. The reviewers had recommended better ways for testing these hypotheses, which we incorporated.**

**We have added the following to the Discussion section (p. 23):**

**“For the sake of transparency, we note that we modified two analytic choices (as described in the Methods section) based on reviewer recommendations. Both our original analyses and the current, reviewer-recommended analyses tested the same hypotheses and yielded similarly null results.”**

*2) The authors use the word “neurotypical” throughout the manuscript (e.g., p. 5, lines 108 and 110), which appears to be a new addition in this revision. It seems that this word is used to refer to individuals who are not high in psychopathic traits; however, “neurotypical” can be interpreted in different ways, so it would be useful to briefly describe what is meant by this word in the authors’ manuscript, in particular.*

**We appreciate the reviewer’s concern about the many interpretations of the word “neurotypical.” To avoid confusion, we have changed all instances of “neurotypical” to “healthy,” which is more commonly used in the literature to describe people who are relatively psychologically healthy.**

*3) On page 5, lines, 109-111, the authors include the following statement that could use a brief elaboration: “Studying high psychopathy people could also advance our understanding of how neurotypical individuals process facial expressions.” Adding a reason why this is the case would help the reader understand the authors’ reasoning and viewpoint.*

**We have revised the sentence on p. 5 to clarify our point:**

**“Identifying aberrant mechanisms in psychopathy could also advance our understanding of how these mechanisms support healthy individuals’ perceptions of affect and emotion in facial expressions.”**

*4) The use of the phrase “high psychopathy people” (e.g., p. 5, lines 109-110) is a bit odd, and saying “individuals high in psychopathic traits” or some other wording might be better.*

**We have changed all instances of “high psychopathy people” to “individuals high in psychopathy.”**

*5) In the Discussion section (p. 24, lines 519-523), the authors mention that their null findings for facial mimicry (even in those low in psychopathic traits) were unexpected, as a past study in a non-incarcerated sample did find spontaneous facial mimicry. It would be helpful to briefly discuss how differences in incarcerated vs. non-incarcerated samples might play a role in the different results.*

**We have added this point to the limitations paragraph in the Discussion (p. 26-27):**

**“It is possible that facial mimicry is less pronounced among incarcerated samples than community samples. In general, incarcerated samples differ from community samples along dimensions that may affect mimicry in response to white faces, including having higher prevalence of mental illness (83) and traumatic brain injury (84), greater proportion of racial and ethnic minority groups (85), and lower socioeconomic status (86). However, Künecke and colleagues found no facial mimicry differences between incarcerated and non-incarcerated individuals (13).”**
